# Exploring Antibiotic Use in the Community: A Household-Based Survey Using the Drug Bag Method in Rural Burkina Faso

**DOI:** 10.3390/antibiotics13090872

**Published:** 2024-09-12

**Authors:** Adélaïde Compaoré, Toussaint Rouamba, Bérenger Kaboré, Jan Jacobs, Koen Peeters Grietens, Salla Sariola

**Affiliations:** 1 Clinical Research Unit of Nanoro, Institut de Recherche en Sciences de la Santé, 11 BP 218 Ouaga CMS 11, Nanoro, Burkina Faso; rouambatoussaint@gmail.com (T.R.); kaboreberenger@gmail.com (B.K.); 2 Socio-Ecological Health Research Unit, Institute of Tropical Medicine Antwerp, 2000 Antwerpen, Belgium; kpeeters@itg.be; 3 Faculté des Sciences Économiques, Sociales, Politiques et de la Communication, UCLouvain Saint-Louis Bruxelles, 1000 Bruxelles, Belgium; 4 Centre de Recherche en Epidémiologie, Biostatistiques et Recherche Clinique, Ecole de Santé Publique, Université Libre de Bruxelles, 1050 Brussels, Belgium; 5 Unit of Bacteriology, Institute of Tropical Medicine Antwerp, 2000 Antwerpen, Belgium; jjacobs@itg.be; 6 School of Tropical Medicine and Global Health, Nagasaki University, 1-12-4 Sakamoto, Nagasaki 852-8523, Japan; 7 Sociology, Faculty of Social Sciences, University of Helsinki, 00014 Helsinki, Finland; salla.sariola@helsinki.fi

**Keywords:** antibiotic use, drug bag method, community, rural, Nanoro, Burkina Faso

## Abstract

In Burkina Faso, there is lack of awareness of antibiotic use at the community level. This study aims to generate information on the commonly used antibiotics along with the reasons for which they have been used in rural Burkina Faso. The drug bag method was employed to collect information from 423 households in the health district of Nanoro. Descriptive analyses were performed using R software version 4.2.1. Of the 33 antibiotics inventoried, amoxicillin tablets and oxytetracycline were the most recognized and used antibiotics. This study indicated that antibiotics were used for a range of health problems in the community, some of which were administered as painkillers. While primary healthcare facilities constituted the primary source of drugs for households (76.8%), informal drug sellers constituted an additional option (61.5%) for community members. This is a significant concern, given that some antibiotics classified as “Watch”—such as norfloxacin—were readily available in these outlets, despite not being included on the country’s Essential Medicines List. This study underscores the necessity of considering the role played by formal providers in the inappropriate use of antibiotics and the importance of understanding the circumstances and logical reasoning underlying communities’ access to and use of antibiotics.

## 1. Introduction

Antibiotics are one of humanity’s medical revolutions, and they have played a pivotal role in combating infectious diseases [1]. In the past two decades, antibiotic use (ABU) in humans has risen in low- and middle-income countries [2,3]. In low- and middle-income countries (LMICs), the use of antibiotics in healthcare for humans is shaped by factors such as inappropriate prescription at healthcare facilities, access to antibiotics from unregulated providers, failure of the healthcare system to meet patients’ needs, and storage of antibiotics for later consumption during common illnesses [4,5,6].

The World Health Organization’s Global Action Plan on Antimicrobial (WHO-GAP) Resistance formulated in 2015 urges the generation of knowledge on antimicrobial resistance (AMR) and antibiotic use in order to optimize the use of antibiotics [7]. Furthermore, as part of its efforts to monitor the use of antibiotics, the WHO has defined three groups of antibiotics for human use—‘Access’, ‘Watch’, and ‘Reserve’ (AWaRe)—based on their clinical importance, specific recommendations for their appropriate use, and their potential for resistance [8,9]. 

In African countries, it is challenging to obtain knowledge on the actual consumption of antibiotics, as the global antibiotic consumption assessment usually reports data on pharmaceutical records, which are known to be either incomplete or unreliable [10]. To the best of our knowledge, there have been only a few studies conducted in Burkina Faso that have investigated the use of antibiotics outside the context of formal healthcare facilities. The current literature on the use of antibiotics documents the prescription patterns observed within healthcare facilities [11,12,13], some of them relying on routinely collected data from primary healthcare facility registers [11], targeting only patients attending clinics [13] and children in particular [11,12]. However, using healthcare facilities’ routine data to study the use of antibiotics cannot provide a complete picture. In LMICs, antibiotics are often purchased over the counter and obtained through informal channels [14]. This implies that relying on healthcare attendance to ascertain antibiotic use may potentially result in a lack of awareness of the antibiotics obtained and used outside of formal healthcare facilities. For instance, a publication on antibiotic use in rural Burkina Faso [13] reported the use of antibiotics prior to hospital attendance, which supports the importance of further investigating antibiotic use at the community level. This survey, conducted in rural setting in Burkina Faso, provides an overview of which antibiotics are used on a routine basis in the community and for what purposes. 

To reach this goal, trustworthy and robust data needed to be generated in order to reflect the conditions prevailing in the research context. To achieve this, this study employed the drug bag method developed by researchers at the London School of Hygiene & Tropical Medicine [15]. The method involves buying locally available antibiotics, so the respondents can determine and classify the antibiotics that are available for them at local sources and to gather information on the specific use of antibiotics. The reason for choosing this approach is due to the existing biases associated with classic surveys on antibiotic use [15,16]. In fact, investigating antibiotic use in households may face the following issues: (i) antibiotics are purchased without prescription, as well as without packets or labels; (ii) people may store antibiotics for later consumption and forget or do not know the type of antibiotic that they are using; and (iii) there is no term for antibiotics in the local language.

Through the study of antibiotic use in the community, antibiotics from different sources can be reported, as can the role played by each of them in the resolution of people’s health concerns. Furthermore, this study serves as an excellent foundation for further research in this area.

## 2. Results

### 2.1. Respondents’ Sociodemographic Characteristics 

Half of the respondents were female (58.6%). The study respondents had a relatively low level of education, as about 57.4% had not attended school. Almost all the respondents (96.7%) were engaged in agricultural activities, with the majority of these respondents engaged in subsistence farming (84.1%). With regard to the possession of antibiotics in households, our investigation revealed a substantial level of drug storage. Indeed, among the 423 households surveyed, almost half of the respondents (48.7%) were found to be storing drugs. The sociodemographic characteristics of the study participants are presented in Table 1.

### 2.2. Sources of Drug Procurement in the Study Households

**Figure 1:**
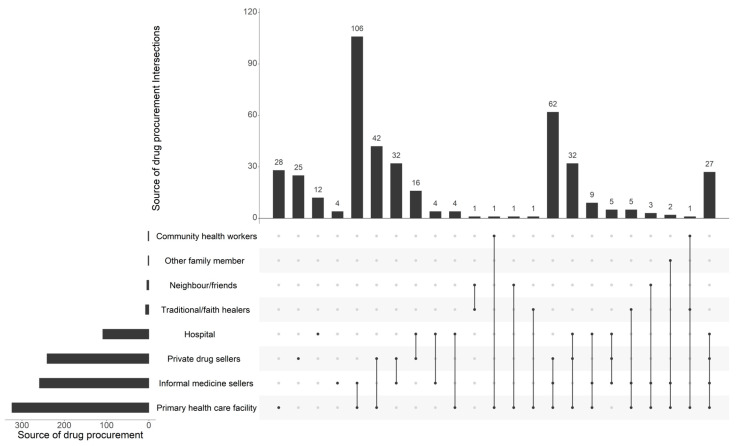
Main sources of drug procurement among the 423 households surveyed.

Our analysis of the sources of drug procurement by household members revealed that approximately three-quarters (76.6%) of respondents obtained their drugs from primary healthcare centers, while 61.2% obtained them through informal vendors, namely at marketplaces. Regarding the private sector, official pharmacies also occupy a significant position in the procurement of antibiotics, as evidenced by the fact that 57% of respondents indicated that they use such pharmacies. However, our analysis revealed that among the respondents, only 28 individuals (out of 423) utilized primary healthcare facilities, while 106 respondents (out of 423) alternatively relied on both primary healthcare facilities and informal medicine sellers. (Figure 1).

### 2.3. Recognition of Antibiotics

A total of 33 antibiotics intended for human healthcare were presented to the respondents in a household setting. The respondents were asked to identify which of the antibiotics they knew or had ever seen, by pointing to them. Each respondent selected a group of antibiotics with which they were familiar. Based on their choices, we found that people were more familiar with amoxicillin (395/423, 93.4%) and oxytetracycline (366/423, 86.5%), followed by ampicillin (295/423, 69.7%), metronidazole (292/423, 69%), and norfloxacin (290/423, 68.6%). The list of recognized antibiotics is presented in Table 2. 

In addition to the quantitative data on recognition, the survey generated further discussions about the recognized antibiotics and, particularly, the local terminologies used in the community, some common antibiotic practices, and reasons for their use. 

Amoxicillin was the only antibiotic that was named by its drug name. The others were identified by their colours:

- Amoxicillin: amoxicillin or ‘toupaye white head’;
- Oxytetracycline: ‘toupaye red head’;
- Norfloxacin: ‘toupaye chinois’ or ‘Chinese toupaye’;
- Ampicillin: ‘toupaye black head’.

Participants were unable to say much about some antibiotics. This was particularly the case for certain antibiotics that were rare in community pharmacies and some of which they were simply unfamiliar with. For example, some men were confused when presented with medicines for pediatric use and called women to provide more details. It was also a challenge to recognize some medicines given in hospitals, for example, as injections, while specialized antibiotics were not easy to name in community pharmacies due to their rarity. Finally, some difficulties in the identification of antibiotics could be attributed to similar shapes and colours. 

### 2.4. Commonly Used Antibiotics

There were 3401 self-reports of antibiotic use in the study area. Amoxicillin, oxytetracycline, ampicillin, metronidazole, and norfloxacin were selected more often than any other drug used. Amoxicillin was reported 367 times (10.8%), oxytetracycline 288 times (8.5%), ampicillin 245 times (7.2%), metronidazole 239 times (7%), and norfloxacin 223 times (6.6%) (Table 3). The top five antibiotics were commonly used, according to the respondents, due to their availability and affordable prices from informal market vendors. 

### 2.5. Health Complaints Leading to ABU

**Figure 2:**
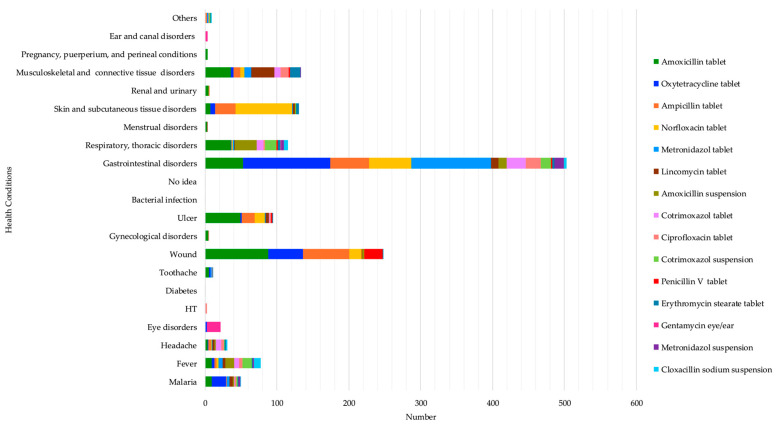
Self-reported health conditions that led to the top 15 antibiotics’ use.

The most reported reasons for taking amoxicillin were wounds at 28.5% (88/309), gastrointestinal disorders at 17.1% (53/309), ulcers at 15.9% (49/309), musculoskeletal and connective tissue disorders at 11.7% (36/309), and respiratory and thoracic disorders at 11.7% (36/309). Oxytetracycline was used to mostly treat gastrointestinal disorders at 57.1% (121/212) and wounds at 22.64% (48/212). Ampicillin was used to treat mostly wounds at 35.32% (65/184), gastrointestinal disorders at 29.34% (54/184), and skin and subcutaneous tissue disorders at 15.21% (28/184). Norfloxacin was mainly taken to treat skin and subcutaneous tissue disorders at 45% (79/180), particularly for the treatment of anal fungus infection in children, as respondents revealed during informal discussions, and gastrointestinal disorders at 32.77% (59/180), while metronidazole was highly used for gastrointestinal disorders (111/132). (See Table S1). These results highlight several health problems that respondents felt required the use of antibiotics, such as gastrointestinal disorders, wounds, skin and subcutaneous tissue disorders, and musculoskeletal and connective tissue disorders. (See Figure 2 below).

## 3. Discussion

The use of antibiotics at the community level is one of the major drivers of antibiotic resistance [17]. This is relevant, as most antibiotics are purchased over the counter and obtained from illegal providers or untrained vendors. To the best of our knowledge, few studies have been conducted in Burkina Faso to investigate antibiotic use outside formal healthcare facilities. The present study aimed at identifying the most used antibiotics in rural communities in Burkina Faso, along with the reasons for their utilization, in order to establish a foundation for a more in-depth investigation towards drivers of the inappropriate use of antibiotics. However, studying antibiotic use among people with low literacy and within a context where there exists no specific term to refer to antibiotics can pose the problem of data accuracy. Due to such complexity, we employed the drug bag method [15], which allows people to physically identify antibiotics and share their knowledge and experiences of them. 

The results of this study demonstrate that the most frequently used antibiotics are in capsule form, and users identify them based on their respective colours. Moreover, findings suggest that antibiotics are employed to address a range of ailments that individuals encounter as part of their everyday lives. 

One of the primary outcomes of this study is the identification of the antibiotics most commonly used in these localities, such as amoxicillin, ampicillin, and metronidazole. This finding is consistent with other research conducted in Burkina Faso [13], Ghana [17], and Tanzania [18], which found that amoxicillin was the most commonly used antibiotic. The widespread use of these antibiotics could be attributed to the fact they belong to the ‘Access group’, which means that they are recommended as first-line treatment options for infections according to the WHO AWaRe framework [9] and because they are registered in the Essential Medicine List (EML) of Burkina Faso [19]. Furthermore, it can be observed that these medicines are also readily available from a variety of over-the-counter retailers as well as informal sellers. During the survey, some metaphorical descriptions of amoxicillin were observed. For instance, amoxicillin was considered “Sagabo” (a local dish in Moore: the local language spoken in the study area) or “Yaaba” (meaning elder or ancestor in Moore). These descriptions corroborate the significant role these medications play in people’s day-to-day lives. Metaphors are being used in the field of anthropology and were considered to have an impact on the way individuals distinguish what is “meaningful and meaningless, valuable and worthless, healthy and sick” [20].

Another finding to consider in this study is the widespread use of certain “Watch antibiotics” such as oxytetracycline and norfloxacin. Yet, these medicines are not included in Burkina Faso’s EML [19], which prompts inquiries regarding their introduction into the community. It can also be hypothesized that their use within communities is attributable, in part, to the fact that they are readily available through informal vendors at affordable prices in marketplaces and without the recommendation of a trained health professional. “Watch antibiotics” are critically important antibiotics that are particularly vulnerable to the emergence of AMR [9]. These findings call for a closer look at the types of medicines available to the general population and the mechanisms that facilitate their availability in the informal sector.

As part of the study objectives, this study allowed the identification of health conditions that lead to the use of antibiotics. Gastrointestinal disorders, wounds, skin and subcutaneous tissue disorders, and musculoskeletal and connective tissue disorders were found to be the top four common health problems that required antibiotic use based on respondents’ answers. The use of antibiotics to treat gastrointestinal disorders, wounds, and skin and subcutaneous tissue disorders necessitates an examination of the hygiene situation that prevails in the daily lives of people in these communities. Evidence suggests that a lack of sanitation and hygiene spreads infectious diseases and, therefore, increases ABU. In the WHO opinion editorial on Water Sanitation and Hygiene (WASH), the regional Director of Southeast Asia explains how poor sanitation and unsafe water are responsible for a range of bacterial infections that increase the use of antibiotics [21]. Consequently, improving hygiene and sanitation can reduce the transmission of AMR and therefore the use of antibiotics [22,23]. Additionally, an anthropological analysis of this situation could be conducted with reference to the work of Chandler and Willis, who interpreted antibiotic use as a “quick fix” for the lack of hygiene in settings of minimized resources [24]. This suggests that antibiotics are used as substitutes for hygiene in non-hygienic conditions within which individuals live and work. In our study setting, open defecation is practiced by half of the total population (58.6%); however, it is particularly common in remote villages such as Nazoanga, where 85.6% of the inhabitants practice open defecation. A thorough exploration of the WASH situation becomes relevant in these settings as they enable better insights into the contextual barriers to improving WASH and the challenges households face in their everyday lives. 

The findings indicated that individuals employed antibiotics to address musculoskeletal and connective tissue disorders. From a biomedical perspective, this can be considered an irrational or knowledge-deficiency-related phenomenon [25]. Nevertheless, this line of reasoning is consistent with a rationalist perspective that views individual behaviour as problematic rather than focusing on structural factors, individual specific circumstances, and reasoning processes [24,26]. In our research setting, people’s primary source of livelihood is derived from agricultural production and income-generating activities. Consequently, individuals are seeking methods to maintain or enhance their health in order to ensure their ability to work. For instance, during the data collection phase, it was documented that amoxicillin and oxytetracycline were frequently combined with alcohol or coffee with the objective of enhancing physical energy and alleviating discomfort, such as joint pain and fatigue. Previous ethnographic research conducted in East Africa supported the idea of antibiotic use in LMICs as “a quick fix for productivity”, where antibiotics are used as a means of not losing wages due to loss of labour [24]. These elements inform the direction of further research, which is necessary to gain a deeper understanding of the underlying motives for antibiotic use in our setting. 

Furthermore, our study prompts us to consider the pathways to antibiotics. Notably, our data indicate that individuals frequently seek medicine from informal market sellers, often referred to as “street drug sellers”. This practice is prevalent and appears to be competitive with the formal healthcare system. Purchasing medicines on the street is a pervasive practice in resource-limited countries [27] and is sometimes recognized as a response to the inadequacies of the health system in contexts where communities cannot afford health costs and access to formal health facilities is limited. Informal drug providers find a way to offer services, as they play a crucial role in in reducing the burden of the healthcare costs (travel costs, drug costs, etc.) and time constraints on people. Healthcare costs have an impact on the inappropriate use of antibiotics, as previous studies in Burkina Faso, Côte d’Ivoire, and other low-income settings have shown [5,6,27,28,29]. Given the important role of informal markets in antibiotic consumption, our findings also raise questions about access to quality medicine in rural communities. Previous studies on antimalarials in Burkina Faso and other LMICs have shown that most substandard antimalarials are found in illicit markets [30,31,32,33,34], which begs the question about the quality of antibiotics as well. 

The drug bag method employed to investigate the use of antibiotics showed that people were likely to choose antibiotics in capsules such as amoxicillin, oxytetracycline, norfloxacin, and ampicillin, which were categorized as ‘toupaye’. The meaning of ‘toupaye’ was unclear to us, as people could not really explain what it meant. In the literature, this term refers to ‘topaye’, from a local dialect in Ghana ‘Twi’, which means ‘throw and burst’ [35]. Yet, other informants believed it was a French distortion of ‘tout passe’ (everything goes away) by Ghanaian drug sellers who had difficulty pronouncing it.

The medicines in question share a common physical appearance in capsule form, which the respondents were able to distinguish based on colour. This recognition phase yielded valuable insights into the level of familiarity with antibiotics among community members. However, it also highlights the potential risk of confusion regarding medicines, as respondents relied on the physical appearance of antibiotics to distinguish them from other medications. Confusing medicines based on their appearance is a common phenomenon in both low- and middle-income countries [36], as well as higher-income settings [37,38]. In our study, certain types of antibiotics looked similar to some non-antibiotic drugs, mainly painkillers, which probably led to their misuse. A study conducted in Ghana on inappropriate antibiotic use raised the issue of confusing antibiotics with painkillers, which are usually in capsules [35]. To address the issue of confusion, some researchers suggest the use of physical appearance tools to improve the identification of oral antibiotics and differentiate them from other commonly sold medicines such as painkillers [36]. This could be a starting point for further reflections on how to reduce the non-essential use of antibiotics. 

There were several limitations to this study. First, the results of the data collected cannot be generalized to the whole country, as the study findings were based on data from a specific rural setting in Burkina Faso. However, these findings provide some baseline information to start some in-depth investigations, as antibiotic use can vary from setting to setting. For example, it allowed for familiarization with local terminologies related to some antibiotics and the potential providers of antibiotics. In addition, it is the most systematic study carried out in the Health and Demographic Surveillance System and in terms of the inventory of available antibiotics from formal and informal sources. It provides an overview of the local use of antibiotics. Finally, our research relied on the self-reporting of antibiotic use, which introduces the problem of recall bias. Nevertheless, despite the respondents’ low level of literacy and the lack of scientific knowledge about antibiotics, we believe that our data are as accurate as possible and that the results are meaningful due to the use of the drug bag method, where participants had the opportunity to physically show the antibiotics. Although this study lists the regularly used antibiotics and identifies where they were obtained, it does not provide information on the frequency or total amount of antibiotics used. 

## 4. Materials and Methods

### 4.1. Study Context

This study is part of an ethnographic study aimed at comprehending antibiotic use at the community level in rural Burkina Faso. The study employed a mixed-methods design with quantitative and qualitative data collection, to elucidate the processes by which antibiotics are prescribed and used, as well as the infrastructural and socioeconomic contexts which shape antibiotic use. This research delves into the relationship between individuals’ socioeconomic circumstances and their antibiotic requirements, exploring how daily activities intersect with antibiotic needs. 

As an initial step in this study, a survey provided essential baseline data, familiarizing researchers with the terminology used in the study setting to refer to particular antibiotics, which informed the subsequent data collection.

### 4.2. Study Sites

**Figure 3:**
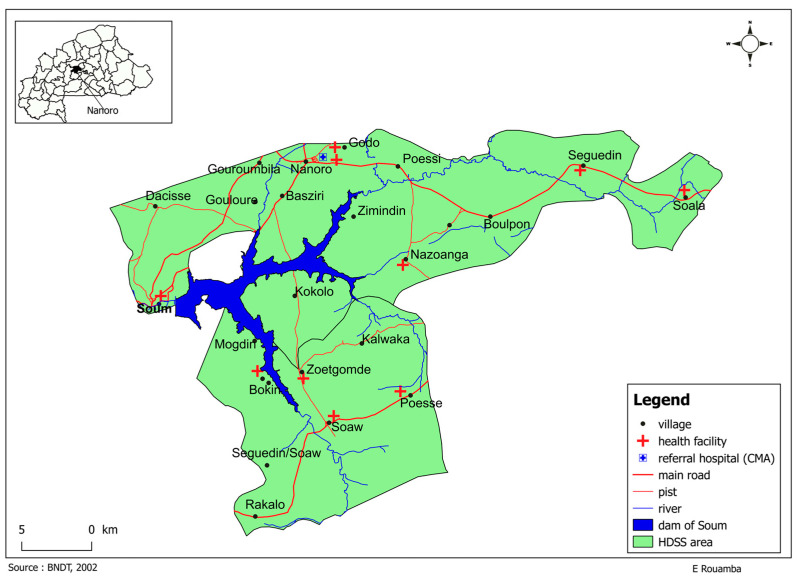
Research site.

The survey was conducted within the Health and Demographic Surveillance System (HDSS) coverage area in the health district of Nanoro, Burkina Faso. (See Figure 3 below). The HDSS was set up in Nanoro in 2009 and covers 24 villages and more than 60,000 inhabitants [39]. Nanoro is in the central west region of Burkina Faso, approximately 85 km from the capital Ouagadougou. It is a typical rural region with an estimated population of 185,160 inhabitants in 2020 [40]. 

The literacy rate is approximately 23% for both men and women [39]. Nanoro health district has 28 primary healthcare facilities and one rural Missionary Hospital (CMA St Camille) with an approximate ratio of 6613 inhabitants per healthcare centre. In 2020, approximately 64% of the population lived within 5 km, 3.2% within 5–10 km, and 32.9% more than 10 km away from a primary healthcare facility [40]. In addition, the region is impoverished, with 53% to 57% of people living with less than USD 0.7 per day [41], which contributes to self-medication strategies. 

In Nanoro, diseases caused by inadequate potable water facilities and poor sanitation are the main causes of health problems [42]. Communicable diseases such as malaria, acute respiratory infections, and fever of unknown origin are leading causes of death among adults and children under five years old [43,44].

### 4.3. Study Participants and Sampling Strategies

Due to the lack of previous studies or pilot studies, which would have allowed us to estimate the prevalence of antibiotic use in the same target population in a rural community, an estimated prevalence of 50% was used to calculate the study sample size. Thus, considering a confidence level of 95%, precision of ± 5%, and 10% maximum missing data, a sample size of 423 households was determined. Therefore, 423 households were randomly selected using the HDSS household sample frame. A proportional allocation of households was performed in accordance with the size of three villages. In order to obtain a contextual description of the phenomenon that this study aimed to explore, three villages were specifically selected on the basis of the HDSS socioeconomic categorization (high, middle, low): Nanoro, Nazoanga, and Gouroumbila.

The proportional size of households was chosen based on the following criteria: (i) the inclusion of all the clusters in a village; (ii) the inclusion of women, men, and young people in the sample. A cluster was defined here as a sub-village composed of a set of locations within the village.

This classification did not aim for a comparative analysis. Instead, the objective was to obtain, as much as possible, a representation of different socioeconomic backgrounds of the population living in the study setting to allow a more realistic description of the ABU.

### 4.4. Data Collection Procedures

We created a database of antibiotics accessible to local people from an inventory of antibiotics available at community drugstores (formal or informal). Hence, antibiotics were purchased in the neighbourhoods either from formal sellers, for instance, in public clinics and authorized private drug stores, or informal sellers at marketplaces. To make our survey as representative as possible, we tried to gather all the antibiotics available to the community members. In the formal context, the purpose of the research was explained to the providers, as some antibiotics could not be sold without a prescription. However, in anticipation of some informal vendors’ reluctance to allow researchers to access their shops, we employed community liaisons or personnel at the Clinical Research Unit of Nanoro (CRUN), who are members of the community. 

With the assistance of a clinician with experience in microbiological research, a list of antibiotics was compiled and categorized according to their active principles and assigned a numeral identifier in alphabetical order. The same number was then allocated to the drug to allow the investigator to tick the corresponding names in the questionnaire.

Amongst the 423 selected households, one respondent was preselected from the HDSS database for an interview. The existing population database permitted the selection of respondents from a range of household members, including fathers, mothers, elders, and young members. The list was communicated to community contact points, whose primary role was to relay information to the HDSS and to facilitate community mobilization. However, during the interviews, it was common to observe that other household members provided responses to certain questions, particularly when the participants were asked to indicate whether they had encountered antibiotics. 

**Figure 4:**
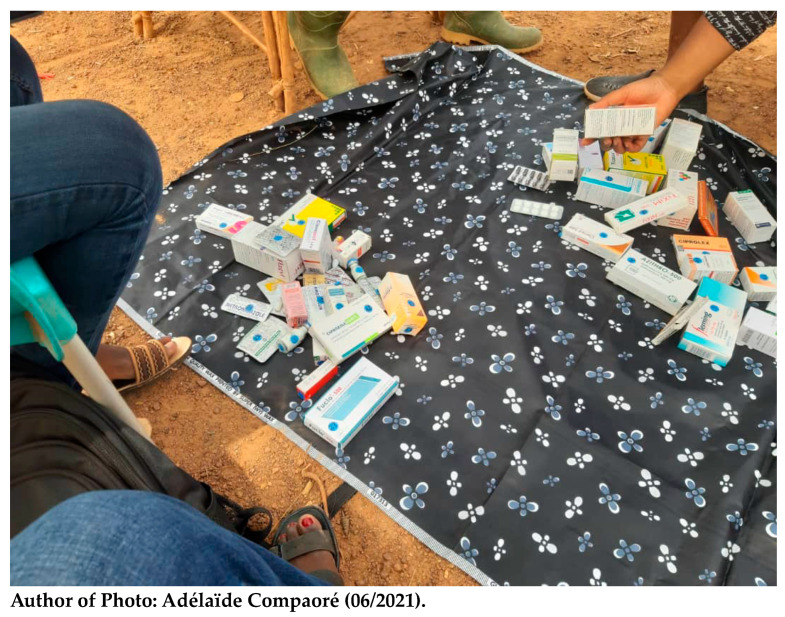
Implementing the drug bag method in Nazoanga.

During the interviews, the medicines were laid out on a cloth and presented one by one to the respondents to provide the following information: which ones they knew or had seen before; which ones they had ever used; which ones they used the most; what health problems required their use. Antibiotics were presented either in the package, in a folio package, or as pills. The packaging and presentation of the antibiotics varied depending on the method of sale. Furthermore, participants were allowed to remove the drugs from the packages and examine them more closely. (See Figure 4 below).

The data were collected via an electronic questionnaire utilizing the software Open Data Kit (ODK) Collect set up v1.4.11. ODK is an open-source suite of tools comprising ODK Collect, an Android-based mobile client that serves as the interface between the user and the underlying form used to collect data. The data were collected in person during the visits to the households by two field assistants with a background in sociology. The data collection period spanned from June to September 2021.

### 4.5. Data Processing and Analysis

Once the data collection was complete, the responses to the survey questions were verified for completeness and consistency. Following this, the verified responses were uploaded to the server (ODK aggregate). Subsequently, the data were then downloaded from the server for the purpose of quality control. All analyses were performed using R software version 4.2.1 (R Development Core Team, R Foundation for Statistical Computing, Vienna, Austria). The descriptive analysis included categorical variables, which are represented as frequencies and percentages, and continuous variables, represented as means with standard deviations.

## 5. Conclusions

This study has the merit of being the first in Burkina Faso to examine antibiotic use at the community level with the drug bag method. The study identified a range of health issues where antibiotics have been used, thereby indicating that within this community, antibiotics are being used for purposes other than their intended function. Moreover, the study revealed that informal medicine vendors represent an important source of antibiotics in the community. This is a significant concern, given that some “Watch” antibiotics, such as norfloxacin, that are part of people’s everyday lives, are readily available through informal channels despite being not included in the country’s Essential Medicine List. This raises concerns about the way antibiotics are obtained within communities, as well as the necessity for further investigation into the circumstances and rationale behind their use.

## Figures and Tables

**Table 1:** Respondents’ sociodemographic characteristics.

		Overall	Nanoro	Nazoanga	Gouroumbila
	N	423	232	125	66
	Gender = Male, n (%)	175 (41.4)	99 (42.7)	52 (41.6)	24 (36.4)
Main occupation, n (%):	Agriculture/farming	271 (64.1)	97 (41.8)	115 (92.0)	59 (89.4)
	Merchant	46 (10.9)	44 (19.0)	2 (1.6)	0 (0.0)
	Pupil/student	27 (6.4)	25 (10.8)	1 (0.8)	1 (1.5)
	Other	19 (4.5)	12 (5.2)	3 (2.4)	4 (6.1)
	Craftsman	17 (4.0)	14 (6.0)	2 (1.6)	1 (1.5)
	Private sector officer	16 (3.8)	16 (6.9)	0 (0.0)	0 (0.0)
	Government officer	11 (2.6)	9 (3.9)	2 (1.6)	0 (0.0)
	Housewife	9 (2.1)	9 (3.9)	0 (0.0)	0 (0.0)
	None	6 (1.4)	5 (2.2)	0 (0.0)	1 (1.5)
	Independent	1 (0.2)	1 (0.4)	0 (0.0)	0 (0.0)
Level of education, n (%):	Uneducated	243 (57.4)	110 (47.4)	94 (75.2)	39 (59.1)
	Incomplete primary school	55 (13.0)	29 (12.5)	19 (15.2)	7 (10.6)
	Informal education	38 (9.0)	17 (7.3)	6 (4.8)	15 (22.7)
	Incomplete secondary	48 (11.3)	45 (19.4)	1 (0.8)	2 (3.0)
	Secondary	17 (4.0)	13 (5.6)	3 (2.4)	1 (1.5)
	Primary school	12 (2.8)	9 (3.9)	1 (0.8)	2 (3.0)
	University	10 (2.4)	9 (3.9)	1 (0.8)	0 (0.0)
Drug storing, n (%):	No	217 (51.3)	105 (45.3)	82 (65.6)	30 (45.5)
	Yes	206 (48.7)	127 (54.7)	43 (34.4)	36 (54.5)

**Table 2:** Antibiotics recognized by the 423 households surveyed.

Antibiotics (N = 423)	n	%
Amoxicillin tablet	398	93.4
Oxytetracycline tablet	369	86.6
Ampicillin tablet	298	70.0
Metronidazole tablet	295	69.2
Norfloxacin tablet	293	68.8
Amoxicillin suspension	247	58.0
Ciprofloxacin tablet	210	49.3
Cotrimoxazole tablet	210	49.3
Penicillin V tablet	204	47.9
Gentamicin eye/ear	203	47.7
Lincomycin tablet	203	47.9
Gentamicin eye	200	46.9
Metronidazole suspension	191	44.8
Cotrimoxazole suspension	181	42.5
Erythromycin stearate tablet	181	42.5
Erythromycin stearate suspension	172	40.4
Cipro eye/ear drop	130	30.5
Cloxacillin sodium suspension	121	28.4
Diloxanide metronidazole suspension	104	24.4
Clavulanic acid tablet	89	20.9
Cefixime suspension	85	20.0
Penicillin injection	82	19.2
Ampicillin sodique	82	19.2
Cloxacillin tablet	76	17.8
Clavulanic acid suspension	76	17.8
Ciprofloxacin tinidazole	62	14.6
Ceftriaxone injection	60	14.1
Cefixime tablet	58	13.6
Azithromycin tablet	47	11.0
Clavulanic acid injection	42	9.9
Neomycin polydexa	40	9.4
Clarithromycin tablet	35	8.2
Flucloxacin tablet	20	4.7

**Table 3:** Distribution of self-reported antibiotic use in the study area (N * = 3401).

Antibiotics	N *	%
Amoxicillin tablet	367	10.8
Oxytetracycline tablet	288	8.5
Ampicillin tablet	245	7.2
Metronidazole tablet	239	7.0
Norfloxacin tablet	223	6.6
Amoxicillin suspension	200	5.9
Co-trimoxazole tablet	157	4.6
Gentamicin eye/ear (drop)	147	4.3
Metronidazole suspension	142	4.2
Ciprofloxacin tablet	135	4.0
Co-trimoxazole suspension	134	3.9
Gentamicin eye (drop)	130	3.8
Penicillin V tablet	127	3.7
Lincomycin tablet	115	3.4
Erythromycin stearate suspension	107	3.1
Erythromycin stearate tablet	88	2.6
Diloxanide furoate metronidazole suspension	74	2.2
Cloxacillin sodium suspension	71	2.1
Ciprofloxacin eye/ear drop (Boncipro)	65	1.9
Amoxicillin/clavulanic acid tablet	48	1.4
Cefixime (Ceficap) suspension	48	1.4
Amoxicillin/clavulanic acid suspension	39	1.1
Penicillin V injection	32	0.9
Cefixime tablet	28	0.8
Ceftriaxone injection	26	0.8
Ampicillin sodium (injection)	23	0.7
Azithromycin tablet	19	0.6
Amoxicillin/clavulanic acid injection (Clavuject)	18	0.5
Ciprofloxacin tinidazole (Ciprozole forte) tablet	17	0.5
Neomycin+Polymyxine B eye drop	15	0.4
Clarithromycin tablet (Clariva)	14	0.4
Cloxacillin tablet	14	0.4
Flucloxacillin tablet	6	0.2

* N Total number of self-declared medicines. Participants may have used more than one medication. Study sample, n = 423.

## Data Availability

Data are contained within the article and Supplementary Materials.

## Supplementary Materials

The following supporting information can be downloaded at: https://www.mdpi.com/article/10.3390/antibiotics13090872/s1, Table S1: Self-reported health conditions which led to antibiotics’ use.

## References

[1] Marquardt R.R., Li S. (2018). Antimicrobial resistance in livestock: Advances and alternatives to antibiotics. Anim. Front..

[2] Klein E.Y., Van Boeckel T.P., Martinez E.M., Pant S., Gandra S., Levin S.A., Goossens H., Laxminarayan R. (2018). Global increase and geographic convergence in antibiotic consumption between 2000 and 2015. Proc. Natl. Acad. Sci. USA.

[3] Van Boeckel T.P., Brower C., Gilbert M., Grenfell B.T., Levin S.A., Robinson T.P., Teillant A., Laxminarayan R. (2015). Global trends in antimicrobial use in food animals. Proc. Natl. Acad. Sci. USA.

[4] Gebeyehu E., Bantie L., Azage M. (2015). Inappropriate use of antibiotics and its associated factors among urban and rural communities of Bahir Dar city administration, northwest Ethiopia. PLoS ONE.

[5] Barker A.K., Brown K., Ahsan M., Sengupta S., Safdar N. (2017). Social determinants of antibiotic misuse: A qualitative study of community members in Haryana, India. BMC Public Health.

[6] Afari-Asiedu S., Oppong F.B., Tostmann A., Ali Abdulai M., Boamah-Kaali E., Gyaase S., Agyei O., Kinsman J., Hulscher M., Wertheim H.F.L. (2020). Determinants of inappropriate antibiotics use in rural central Ghana using a mixed methods approach. Front. Public Health.

[7] WHO (2015). Global Action Plan on Antimicrobial Resistance.

[8] Zanichelli V., Sharland M., Cappello B., Moja L., Getahun H., Pessoa-Silva C., Sati H., van Weezenbeek C., Balkhy H., Simão M. (2023). The WHO AWaRe (Access, Watch, Reserve) antibiotic book and prevention of antimicrobial resistance. Bull. World Health Organ..

[9] WHO (2022). The WHO AWaRe (Access, Watch, Reserve) Antibiotic Book.

[10] Frost I., Kapoor G., Craig J., Liu D., Laxminarayan R. (2021). Status, challenges and gaps in antimicrobial resistance surveillance around the world. J. Glob. Antimicrob. Resist..

[11] Sié A., Ouattara M., Bountogo M., Dah C., Compaoré G., Boudo V., Lebas E., Brogdon J., Nyatigo F., Arnold B.F. (2021). Indication for antibiotic prescription among children attending primary healthcare services in rural Burkina Faso. Clin. Infect. Dis..

[12] Sié A., Coulibaly B., Adama S., Ouermi L., Dah C., Tapsoba C., Bärnighausen T., Kelly J.D., Doan T., Lietman T.M. (2019). Antibiotic prescription patterns among children younger than 5 years in Nouna district, Burkina Faso. Am. J. Trop. Med. Hyg..

[13] Valia D., Ingelbeen B., Kaboré B., Karama I., Peeters M., Lompo P., Vlieghe E., Post A., Cox J., de Mast Q. (2022). Use of WATCH antibiotics prior to presentation to the hospital in rural Burkina Faso. Antimicrob. Resist. Infect. Control.

[14] World Health Organisation (2023). Antimicrobial Resistance: Key Facts. https://www.who.int/news-room/fact-sheets/detail/antimicrobial-resistance.

[15] Dixon J., MacPherson E., Manyau S., Nayiga S., Khine Zaw Y., Kayendeke M., Nabirye C., Denyer Willis L., de Lima Hutchison C., Chandler C.I.R. (2019). The ‘Drug Bag’ method: Lessons from anthropological studies of antibiotic use in Africa and South-East Asia. Glob. Health Action.

[16] Das J., Hammer J., Sánchez-Paramo C. (2012). The impact of recall periods on reported morbidity and health seeking behavior. J. Dev. Econ..

[17] Kretchy J.-P., Adase S.K., Gyansa-Lutterodt M. (2021). The prevalence and risks of antibiotic self-medication in residents of a rural community in Accra, Ghana. Sci. Afr..

[18] Mabilika R.J., Mpolya E., Shirima G. (2022). Prevalence and predictors of self-medication with antibiotics in selected urban and rural districts of the Dodoma region, Central Tanzania: A cross-sectional study. Antimicrob. Resist. Infect. Control.

[19] Ministry of Health of Burkina Faso (2020). Liste Nationale des Medicaments Essentiels et Autres Produits de Santé.

[20] van der Geest S., Whyte S.R. (1989). The charm of medicines: Metaphors and metonyms. Med. Anthropol. Q..

[21] Poonam Khetrapal Singh (2017). Water, Sanitation, and Hygiene: An Essential Ally in a Superbug Age. WHO..

[22] Pinto Jimenez C.E., Keestra S., Tandon P., Cumming O., Pickering A.J., Moodley A., Chandler C.I.R. (2023). Biosecurity and water, sanitation, and hygiene (WASH) interventions in animal agricultural settings for reducing infection burden, antibiotic use, and antibiotic resistance: A One Health systematic review. Lancet Planet. Health.

[23] Bürgmann H., Frigon D., Gaze W.H., Manaia C.M., Pruden A., Singer A.C., F Smets B., Zhang T. (2018). Water and sanitation: An essential battlefront in the war on antimicrobial resistance. FEMS Microbiol. Ecol..

[24] Denyer Willis L., Chandler C. (2019). Quick fix for care, productivity, hygiene and inequality: Reframing the entrenched problem of antibiotic overuse. BMJ Case Rep..

[25] Mboya E.A., Davies M.L., Horumpende P.G., Ngocho J.S. (2020). Inadequate knowledge on appropriate antibiotics use among clients in the Moshi municipality Northern Tanzania. PLoS ONE.

[26] Rodrigues C.F. (2020). Self-medication with antibiotics in Maputo, Mozambique: Practices, rationales and relationships. Palgrave Commun..

[27] Bloom G., Standing H., Lucas H., Bhuiya A., Oladepo O., Peters D.H. (2011). Making health markets work better for poor people: The case of informal providers. Health Policy Plan..

[28] Angbo-Effi K.O., Kouassi D.P., Yao G.H.A., Douba A., Secki R., Kadjo A. (2011). Determinants of street drug use in urban areas. Sante Publique.

[29] Dagrou A., Chimhutu V. (2022). I buy medicines from the streets because I am poor: A qualitative account on why the informal market for medicines thrive in Ivory Coast. Inquiry.

[30] Tipke M., Diallo S., Coulibaly B., Störzinger D., Hoppe-Tichy T., Sie A., Müller O. (2008). Substandard anti-malarial drugs in Burkina Faso. Malar. J..

[31] Hoellein L., Kaale E., Mwalwisi Y.H., Schulze M.H., Vetye-Maler C., Holzgrabe U. (2022). Emerging antimicrobial drug resistance in Africa and Latin America: Search for reasons. Risk Manag. Healthc. Policy.

[32] WHO (2017). A Study on Public Health and Socioeconomic Impact of Substandard and Falsified Medicines.

[33] Gnegel G., Hauk C., Neci R., Mutombo G., Nyaah F., Wistuba D., Häfele-Abah C., Heide L. (2020). Identification of falsified chloroquine tablets in Africa at the time of the COVID-19 pandemic. Am. J. Trop. Med. Hyg..

[34] WHO (2018). Substandard and Falsified Medical Products—Fact Sheets. World Health Organization..

[35] Afari-Asiedu S., Hulscher M., Abdulai M.A., Boamah-Kaali E., Asante K.P., Wertheim H.F.L. (2020). Every medicine is medicine; exploring inappropriate antibiotic use at the community level in rural Ghana. BMC Public Health.

[36] Monnier A.A., Do N.T.T., Asante K.P., Afari-Asiedu S., Khan W.A., Munguambe K., Sevene E., Tran T.K., Nguyen C.T.K., Punpuing S. (2023). Is this pill an antibiotic or a painkiller? Improving the identification of oral antibiotics for better use. Lancet Glob. Health.

[37] Mira J.J., Ortiz L., Lorenzo S., Royuela C., Vitaller J., Pérez-Jover V. (2014). Oversights, confusions and misinterpretations related to self-care and medication in diabetic and renal patients. Med. Princ. Pract..

[38] Tranchard F., Gauthier J., Hein C., Lacombe J., Brett K., Villars H., Sallerin B., Montastruc J.L., Despas F. (2019). Drug identification by the patient: Perception of patients, physicians and pharmacists. Therapie.

[39] Derra K., Rouamba E., Kazienga A., Ouedraogo S., Tahita M.C., Sorgho H., Valea I., Tinto H. (2012). Profile: Nanoro health and demographic surveillance system. Int. J. Epidemiol..

[40] Ministry of Health of Burkina Faso (2021). Annuaire Statistique 2020.

[41] Zida Y., Kambou S.H., Cartographie de la Pauvreté et des Inégalités au Burkina Faso (2014). PNUD. https://www.undp.org/fr/burkina-faso/publications/cartographie-de-la-pauvrete-et-des-inegalites-au-burkina-faso.

[42] Tinto H., Valea I., Sorgho H., Tahita M.C., Traore M., Bihoun B., Guiraud I., Kpoda H., Rouamba J., Ouédraogo S. (2014). The impact of clinical research activities on communities in rural Africa: The development of the Clinical Research Unit of Nanoro (CRUN) in Burkina Faso. Malar. J..

[43] Ministry of Health of Burkina Faso (2017). Annuaire Statistique 2016.

[44] Maltha J., Guiraud I., Kaboré B., Lompo P., Ley B., Bottieau E., Van Geet C., Tinto H., Jacobs J. (2014). Frequency of severe malaria and invasive bacterial infections among children admitted to a rural hospital in Burkina Faso. PLoS ONE.

